# *Kazachstania slooffiae* Fungemia: A Case Report and Literature Review on an Emerging Opportunistic Pathogen in Humans

**DOI:** 10.1093/ofid/ofaf209

**Published:** 2025-04-04

**Authors:** Sarah N Fortna, David M Aronoff, Andrew T Dysangco

**Affiliations:** Department of Medicine, Indiana University School of Medicine, Indianapolis, Indiana, USA; Department of Medicine, Indiana University School of Medicine, Indianapolis, Indiana, USA; VA Atlanta Healthcare System, Decatur, Georgia, USA

**Keywords:** *Kazachstania slooffiae*, candida, fungemia, case report, mycoses

## Abstract

**Background:**

We report a case of *Kazachstania slooffiae* fungemia in a 77-year-old immunocompromised male with gastrointestinal abnormalities including achalasia, gastroparesis, and prior esophagectomy. He presented with sepsis, gastric ischemia, and pleural effusion. To date, only 2 prior cases of *Kazachstania slooffiae* infection have been reported, both involving nonbloodstream infections.

**Methods:**

We reviewed the existing literature and compared clinical, diagnostic, and treatment characteristics across all identified cases.

**Results:**

*Kazachstania slooffiae* has been identified in critically ill, immunocompromised patients with gastrointestinal anatomic abnormalities, underlying malignancy, and disease. Diagnosis relies on advanced methods such as matrix-assisted laser desorption/ionization time-of-flight mass spectrometry and DNA sequencing. Antifungal treatments (caspofungin, fluconazole, micafungin) have resulted in favorable outcomes.

**Conclusions:**

This review highlights *Kazachstania slooffiae* as a rare opportunistic pathogen and provides the first evidence for *Kazachstania slooffiae* as a bloodstream pathogen in humans. Prompt identification and antifungal therapy are critical for managing infections.

The incidence of uncommon invasive fungal infections (IFIs), that is, those caused by non-*albicans Candida* species, is increasing [1]. *Kazachstania slooffiae* (formerly *Candida slooffiae*) presents a further challenge due to limited knowledge of its role as a human pathogen [1–4].

The yeast genus *Kazachstania* was first described over 50 years ago by Zubkova, with the isolation of *Kazachstania viticola* from fermenting grapes in Kazakhstan [5]. Members of this genus are typically found in diverse environments, including animal gastrointestinal (GI) tracts, soil, and decaying organic matter [6]. *Kazachstania* species also play a role in winemaking and traditional French bread-making [7]. *K. slooffiae* is a member of the *K. telluris* complex and has been isolated most frequently from horses and healthy swine. It is suspected to be a commensal of the porcine intestinal microbiome [8–10].

Although *K. slooffiae* has been identified as a commensal organism in other animals, its role as a human pathogen is underexplored. To date, only nonbloodstream infections have been reported in the literature. To our knowledge, this is the first documented case of *K. slooffiae* fungemia in a human.

## METHODS

A comprehensive literature search was conducted between December 2024 and January 2025 in PubMed and Scopus to identify published cases of human infections caused by *K. slooffiae.* The search was performed using the following search terms: “*Kazachstania*” AND “human” and “*Candida slooffiae*” AND “human.” Results were filtered to include only English-language publications. This search yielded 46 references, which were exported for further screening. The references retrieved from PubMed were imported into Rayyan.ai, an online tool for collaborative and systematic review screening. The inclusion and exclusion criteria were as follows:

- Inclusion criteria: case reports or studies describing human infections caused by *K. slooffiae* or *Candida slooffiae.*
- Exclusion criteria: nonhuman studies, reviews without primary case data, and non-English-language studies.

Two reviewers independently screened titles and abstracts in Rayyan.ai to ensure that only relevant studies were included. Discrepancies were resolved through discussion. After screening, 2 case reports were included in the final analysis. Key data points analyzed included patient demographics, clinical presentation, infection source, diagnostics, treatment, and outcomes.

## CASE REPORT

A 77-year-old male presented to the emergency department with fatigue, shortness of breath, abdominal pain, nausea, and vomiting. He had a history of achalasia with gastroparesis requiring esophagectomy with gastric pull-through and jejunostomy tube (J-tube) placement. He was J-tube dependent and severely chronically malnourished, evidenced by >10% weight loss over the preceding 6 months and significant loss of muscle mass and subcutaneous fat. His immunocompromised state was further exacerbated by *Enterococcus faecalis* endocarditis 4 months prior, which required bioprosthetic aortic valve replacement due to subsequent heart failure. This was complicated by recurrent pleural effusion managed with an indwelling pleural catheter. The patient had no farm or other animal exposures.

On admission the patient had severe sepsis with hypotension, tachypnea, and sinus tachycardia. Physical examination revealed moderately increased work of breathing, rales in the right chest, and bowel sounds with decreased breath sounds at the right base. There was a pleural catheter with no surrounding erythema or purulence. The left lung was clear to auscultation, and a 2/6 systolic ejection murmur was noted over the precordium.

**Figure 1.**
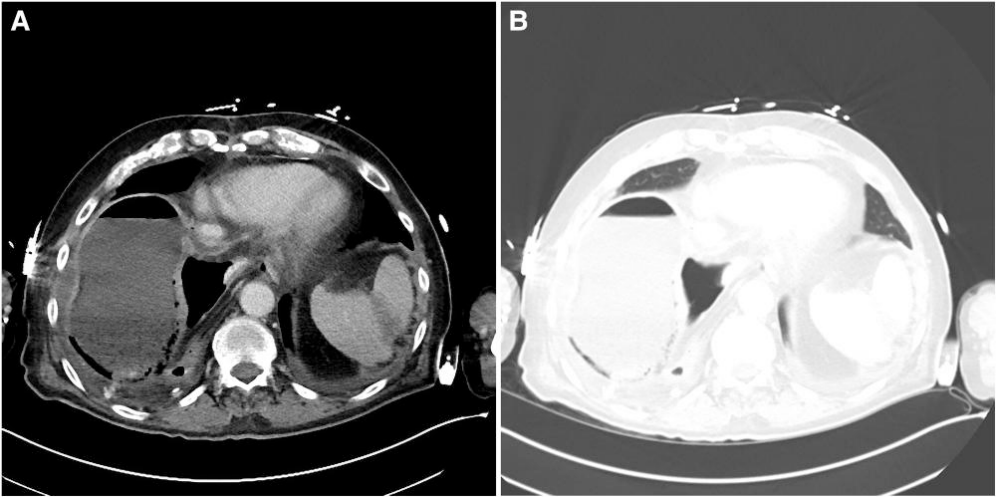
*A*, Axial contrast-enhanced CT at the level of lung bases. The patient has a remote history of esophagectomy with gastric pull-through, lending to his altered anatomy. Foci of gas are seen within the gastric wall, consistent with gastric pneumatosis. *B*, Axial contrast-enhanced CT viewed in the lung window to better visualize tissue density and distinguish air-filled space from fluid-filled space. Abbreviation: CT, computed tomography.

**Figure 2.**
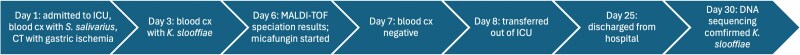
Delineated timeline of patient's clinical progression. Abbreviations: CT, computed tomography; ICU, intensive care unit; MALDI-TOF, matrix-assisted laser desorption/ionization time-of-flight.

The patient was started on broad-spectrum antibiotics and admitted to the medical intensive care unit (ICU). Computed tomography (CT) scans showed gastric distension with pneumatosis and portal venous gas, as well as consolidation in the right middle and upper lobes (Figures 1 and 2). Vancomycin and piperacillin-tazobactam were started empirically. A nasogastric tube was placed for gastric decompression. His pleural drain was replaced with a temporary chest tube for source control. Blood cultures from admission grew *Streptococcus salivarius* in both sets. Pleural fluid culture grew *Stenotrophomonas maltophilia, Klebsiella oxytoca,* and *Corynebacterium jeikeium*.

On day 3 of admission, repeat blood cultures grew *Candida* species in 2 aerobic bottles, which the Vitek 2 system failed to speciate. However, matrix-assisted laser desorption/ionization time-of-flight mass spectrometry (MALDI-TOF MS) identified the organism as *Candida* (*Kazachstania*) *slooffiae.* This was confirmed via DNA sequencing through LabCorp 1 month later. An echinocandin (intravenous micafungin 100 mg daily) was initiated empirically for fungemia based on the 2016 Infectious Diseases Society of America candidemia guidelines. Minimum inhibitory concentrations (MICs) for antifungals were not obtained. Repeat blood cultures after the initiation of micafungin were negative. Transthoracic echocardiogram and cardiac CT showed no evidence of vegetations or other new cardiac complications. A transesophageal echocardiogram could not be obtained due to his anatomy. Ophthalmology consultation was not necessary due to absence of visual or ocular complaints. Due to uncertainty regarding yeast identification and sensitivities and the possibility of endocarditis based on 2023 Modified Duke Criteria, micafungin was continued for 6 weeks. Figure 3 illustrates the timeline of the patient's presentation.

Antibiotics were tailored to cover the patient's bacteremia, possible bioprosthetic valve endocarditis, empyema, and ischemic bowel. Piperacillin-tazobactam was de-escalated to ampicillin-sulbactam, followed by amoxicillin-clavulanate over 4 weeks. The patient also received minocycline and trimethoprim-sulfamethoxazole to target *S. maltophilia* and vancomycin for *C. jeikeium*. The vancomycin was extended to 6 weeks’ duration due to possible viridans streptococcal endocarditis.

The patient gradually improved after initiation of anti-infectives, gastric decompression, and chest tube insertion. No surgical intervention was required. Two days after starting micafungin, he was transferred out of the ICU. After a 3-week hospitalization, he was discharged in stable condition. At 8-month follow-up, the patient reported that he had been regaining his strength, recovering well, had no progression of difficulty with tube feedings, and no relapse of fungal infection was found. No further antifungal prophylaxis was required at that time.

## CASE REVIEW AND DISCUSSION

We identified 2 other reported cases of human IFIs caused by *K. slooffiae* (Table 1). Including our case, all 3 patients were male and had either current or previous gastroesophageal disease. This suggests that gastroesophageal pathology may predispose individuals to *K. slooffiae* IFI (Table 2). Only 1 case involved farm animal exposure, related to the patient's occupation. The organism caused a mediastinitis and empyema as a complication of ischemic perforated colon surgery, an esophagitis, and a fungemia from gastric ischemia [9–11]. Table 1 summarizes the key clinical and diagnostic features of the reported cases, while Table 2 highlights the common underlying GI conditions present in all 3 patients.

**Table 1.** Comparison of Published Cases of *Kazachstania slooffiae* With Our Case

Study (year)	Mercier et al. (2021)	Gallotti et al. (2023)	Fortna et al. (2025)
Age/sex	50-year-old male	80-year-old male	77-year-old male
Medical history	Adenocarcinoma s/p esophagectomy with neoadjuvant chemotherapy	Prostate cancer, iron deficiency anemia, progressive liquid dysphagia (worsening)	Achalasia with gastroparesis s/p esophagectomy, recent endocarditis s/p bioprosthetic aortic valve replacement
Clinical presentation	Mediastinitis as complication of ischemic perforated colon surgery	Esophagitis	Pleural effusion, gastric ischemia, fungemia
Sample type	Pleural fluid	Esophageal biopsy	Blood cultures
Diagnostic methods	Culture, MALDI-TOF MS, DNA sequencing	PAS stain, MALDI-TOF MS, DNA sequencing	Culture, MALDI-TOF MS, DNA sequencing
Antifungal treatment	Caspofungin ×6 wk	Fluconazole ×21 d	Micafungin ×6 wk
Outcome	Resolution	Resolution	Resolution

Abbreviations: CT, computed tomography; ICU, intensive care unit; MALDI-TOF, matrix-assisted laser desorption/ionization time-of-flight; MS, mass spectrometry; PAS, periodic acid-Schiff.

**Table 2.** Possible Risk Factors in *Kazachstania slooffiae* Infections

Study (year)	Mercier et al. (2021)	Gallotti et al. (2023)	Fortna et al. (2025)
Gastrointestinal abnormality	Gastroesophageal adenocarcinoma s/p esophagectomy, ischemic colonic perforation	Dysphagia, esophagitis	Achalasia with gastroparesis s/p esophagectomy with gastric pull-through and jejunostomy tube, gastric ischemia
Immunocompromised state	Adenocarcinoma, chemotherapy	Prostate cancer, iron deficiency anemia	Recent infections, malnourishment
Advanced age	50 y	80 y	77 y
Sepsis or severe infection	Septic shock, ARDS	No sepsis	Sepsis, fungemia, recent infective endocarditis
Fungal entry via GI tract	Colonic perforation and subsequent mediastinal contamination	Esophageal colonization	GI translocation from gastric ischemia

Abbreviations: ARDS, acute respiratory distress syndrome; GI, gastrointestinal.

Given that GI abnormalities (eg, surgery, ischemia) were observed in all 3 cases, it is plausible that *K. slooffiae* is a part of the human GI microbiome in some individuals, although further studies are needed to explore its potential as a colonizer. The mechanism by which it translocates to the bloodstream remains unclear but is most likely due to a disruption in the mucosal barrier. Only 1 case involved animal exposure, and the clinical relevance of this exposure in relation to the infection remains uncertain. Human GI abnormalities appear to be the more likely contributing factor, and in our case gastric ischemia likely facilitated translocation.

All 3 cases were identified through cultures of clinical specimens, speciated by MALDI-TOF MS, and confirmed by DNA sequencing [9, 10]. Although MALDI-TOF MS accurately identified *K. slooffiae* in all 3 cases, there is a case report of *K. bovina* bacteremia that was misidentified by MALDI-TOF MS as *K. slooffiae,* suggesting that it may have difficulty distinguishing between members of the *K. telluris* complex [12]. Given the potential for misidentification within the *Kazachstania* genus, we recommend that clinical laboratories use a combination of MALDI-TOF MS with other diagnostic methods such as DNA sequencing to confirm the diagnosis of rare pathogens like *K. slooffiae*.

There are no established breakpoints nor treatment guidelines for *K. slooffiae,* and antifungal susceptibility testing remains challenging due to poor growth conditions for the organism [10, 11]. A study using 26 *K. slooffiae* isolates from piglets found no resistance to fluconazole, though sensitivity determination was not described [8]. In the reported human cases, fluconazole was used for esophagitis, while echinocandins were used for empyema and in our fungemia case. All 3 cases responded well to antifungal therapy, with favorable clinical outcomes [9, 10]. With limited data, fluconazole may be suitable for low-risk infections, with careful monitoring and MIC determination. For more severe infections, echinocandins are better alternatives.

The small number of reported cases, as well as the lack of antifungal susceptibility data, presents a significant limitation to understanding *K. slooffiae* as a human pathogen. Future case series would be valuable to gather more data on this rare pathogen. Further studies on antifungal susceptibility testing, optimal treatment regimens, and the pathogenic mechanisms of *K. slooffiae* are needed.

## CONCLUSIONS

While the overall incidence remains low, the growing recognition of rare opportunistic yeast, such as *K. slooffiae,* is crucial for improving diagnostic and therapeutic strategies. Diagnosis requires advanced methods such as MALDI-TOF MS and DNA sequencing. Antifungal therapies, including caspofungin, fluconazole, and micafungin, have been effective in reported cases. The clinical course and treatment outcomes in our case contribute valuable insights into the management of fungemia caused by uncommon pathogens.
